# Substance Use Disorders and other Mental Health Disorders associated with sexualized intravenous sbustance use (slamsex)

**DOI:** 10.1192/j.eurpsy.2023.823

**Published:** 2023-07-19

**Authors:** J. Curto Ramos, P. Barrio, L. Ibarguchi, A. García, J. Garde, A. Oliva, H. Dolengevich Segal

**Affiliations:** 1 Department of Psychiatry, Clinical Psychology and Mental Health, La Paz University Hospital, Madrid, Spain, Hospital la Paz; 2 NGO Apoyo Positivo, Madrid; 3 Department of Psychiatry, Clinical Psychology and Mental Health, Hospital del Henares, Coslada, Spain

## Abstract

**Introduction:**

The intentional use of drugs before or during sexual intercourse (chemsex) is a phenomenon of special importance in the MSM (men who have sex with men) population due to its impact on mental, physical and sexual health.

**Objectives:**

The objective of this study was to compare the psychopathological characteristics between users whith sexualized intravenous substance use (slamsex) versus those who did not slamsex attended by the non-govenrmental organization Apoyo Positivo in the program “Sex, Drugs and You”.

**Methods:**

A cross-sectional descriptive analysis of a sample of users attended by the non-govenrmental organization Apoyo Positivo in the program “Sex, Drugs and You” was performed.

**Results:**

230 participants were included. Slam was associated with higher risk of having and anxiety or depressive disorder, suicidal ideation, induced psychosis and suicidal behavior, the differences were statistically significant (p<0.05) in all cases.

**Conclusions:**

Slamsex is usually reported in our sample. Substance use disorders in slam users are usually associated with other mental disorders. This challenge requires adapt the therapeutical interventions of the professionals who work with patients with chemsex practices, specially with those who practice slamsex.

**Disclosure of Interest:**

None Declared

